# Biological Activity of Flavonoids and Rare Sesquiterpene Lactones Isolated From *Centaurea ragusina* L.

**DOI:** 10.3389/fphar.2018.00972

**Published:** 2018-08-22

**Authors:** Ulrike Grienke, Sandra Radić Brkanac, Valerija Vujčić, Ernst Urban, Siniša Ivanković, Ranko Stojković, Judith M. Rollinger, Juran Kralj, Anamaria Brozovic, Marijana Radić Stojković

**Affiliations:** ^1^Department of Pharmacognosy, University of Vienna, Vienna, Austria; ^2^Department of Biology, Faculty of Science, University of Zagreb, Zagreb, Croatia; ^3^Department of Pharmaceutical Chemistry, University of Vienna, Vienna, Austria; ^4^Division of Molecular Medicine, Ruđer Bošković Institute, Zagreb, Croatia; ^5^Division of Molecular Biology, Ruđer Bošković Institute, Zagreb, Croatia; ^6^Division of Organic Chemistry and Biochemistry, Ruđer Bošković Institute, Zagreb, Croatia

**Keywords:** *Centaurea*, sesquiterpene lactones, DNA, cell viability, toxicity, glutathione

## Abstract

The endemic Croatian species *Centaurea ragusina* L., like other species from the genus *Centaurea*, has been traditionally used in Croatia as an antibacterial agent and for the treatment of gastrointestinal and urogenital disorders. In several chromatographic steps, three flavonoids and three sesquiterpene lactones (STLs) were isolated and identified from the most active fractions of the ethanol extract. Two STLs, one for which we created the trivial name ragusinin, and hemistepsin A are here reported for the first time as constituents of the genus *Centaurea*. All six compounds were screened for their effect on several tumor and one normal cell lines. Among them, ragusinin showed the best bioactivity and high specificity to affect tumor murine SCCVII, human HeLa and Caco-2 cell lines, but not the viability of normal V79 fibroblasts. Due to these characteristics the action of ragusinin was investigated in more detail. Since DNA is the primary target for many drugs with antibacterial and anticancer activity, we studied its interaction with ragusinin. Rather moderate binding affinity to DNA excluded it as the primary target of ragusinin. Due to the possibility of STL interaction with glutathione (GSH), the ubiquitous peptide that traps reactive compounds and other xenobiotics to prevent damage to vital proteins and nucleic acids, its role in deactivation of ragusinin was evaluated. Addition of the GSH precursor N-acetyl-cysteine potentiated the viability of HeLa cells, while the addition of GSH inhibitor L-buthionine sulfoximine decreased it. Moreover, pre-treatment of HeLa cells with the inhibitor of glutathione-S-transferase decreased their viability indicating the detoxifying role of GSH in ragusinin treated cells. Cell death, derived by an accumulation of cells in a G2 phase of the cell cylce, was shown to be independent of poly (ADP-ribose) polymerase and caspase-3 cleavage pointing toward an alternative cell death pathway.

## Introduction

The genus *Centaurea* (Asteraceae) represents an attractive source for bioactive secondary metabolites such as sesquiterpene lactones (STLs), flavonoids, lignans, and their glycosides (Khammar and Djeddi, 2012). A number of therapeutic effects against microbial infections, gastrointestinal disorders, and urogenital ailments have been attributed to *Centaurea* species in Croatian folk medicine and worldwide (Pahlow, 1989; Ayad et al., 2012; Politeo et al., 2012).

Our recent study on the phytochemical and bioactive profile of non-volatile constituents of *Centaurea ragusina* L., an endemic Croatian halophytic species (Radić et al., 2013), indicated the strong potential for obtaining bioactive compounds from the leaf ethanol extract (CRE) (Vujčić et al., 2017).

In continuation of this previous study, the aim was to investigate the biological activity of all isolated compounds, namely the interaction with DNA, the antibacterial activity against Gram-positive (*Staphylococcus aureus*) and Gram-negative bacteria (*Acinetobacter baumannii*) (Lee et al., 2007; Ćurković-Perica et al., 2015) and anticancer activity against a panel of murine and human cancer cells.

Due to its high biological significance, DNA is the primary target for many drugs with antibacterial and anticancer activity. Small organic molecules can bind to DNA by means of a non-specific, electrostatic binding along the DNA backbone, a specific groove binding and intercalation or can form crosslinks with DNA strands and induce cleavage of the DNA backbone (Demeunynck et al., 2002; Sangeetha Gowda et al., 2014).

STLs are known to bind covalently to sulfhydryl groups of enzymes and other functional proteins by Michael type addition of their electrophilic α, β-unsaturated carbonyl structures. It is believed that most of STL biological effects are due to their reaction with biological nucleophiles such as GSH (Kupchan et al., 1971; Picman, 1986; Schmidt, 1999). Among many roles of GSH in the cell, the most important one seems to be the removal of reactive species and elimination of xenobiotic compounds. The last one can be accomplished through conjugation with GSH followed by secretion of adducts from the cell (Boyland and Chasseaud, 1969).

Here, the aim was to study the activity of the most bioactive compound in more detail, which included the interaction with DNA as the potential primary target and the interaction with GSH and its impact on cytotoxicity, cell cycle and cell death.

## Materials and Methods

All safety precautions were taken when working with chemicals reagents used in the experiments.

### General Experimental Procedures

1D and 2D NMR experiments were performed on an Avance 500 MHz instrument equipped with cryoprobe (Bruker, Billerica, MA, United States). The samples were measured in MeOD and DMSO-d6, respectively (calibrated to the residual non-deuterated solvent signals). HR-ESI-MS analyses were performed on a maXis HD ESI-Qq-TOF mass spectrometer (Bruker Daltonics, Bremen, Germany). The ESI ion source was operated as follows: capillary voltage: 2.0 to 4.5 kV (individually optimized), nebulizer: 0.4 bar (N_2_), dry gas flow: 4 L min^-1^ (N_2_) and dry temperature: 200°C, scanning range, *m/z* 50–1550. For each isolated compound, fragment ion spectra of the [M+H]^+^, the [M+Na]^+^ and either the [M-H] ^-^ or the [M+HCOO]^-^ ion were recorded. The sum formulas of the ions were determined using Bruker Compass DataAnalysis 4.2 based on the mass accuracy (Δ*m/z* ≤ 2 ppm for MS1 and ≤ 3 ppm for MS/MS) and isotopic pattern matching (SmartFormula algorithm).

Column chromatography (CC) was performed using Merck silica gel 60 (40–63 μm) and Pharmacia Sephadex LH-20 (20–100 μm). The fractions obtained from all chromatographic steps were analyzed by TLC (mobile phase: CH_2_Cl_2_-EtOAc (85:15), *n*-hexane-EtOAc-CH_3_COOH (6:3:1), or *n*-hexane-EtOAc (8:2); stationary phase: Merck silica gel 60 PF_254_, detected with staining reagents vanillin/H_2_SO_4_ at vis, UV_254_, and UV_366_). HPLC was performed on a Shimadzu UFLC-XR instrument (Kyoto, Japan) with a photodiode array detector (DAD). LC-parameters: stationary phase: Phenomenex Gemini-NX (C18), 150 mm × 3.00 mm, 5 μm; temperature: 35°C; mobile phase: water with 0.1% formic acid (A); acetonitrile (B); flow rate 0.4 mL/min; UV detection wavelength: 275 nm; injection volume: 10 μL; gradient: 80/20 A/B in 5 min to 70/30 A/B, then within 20 min to 50/50 A/B and within another 2 min to 2/98, followed by a 5 min column wash (2A/98B) and a re-equilibration period of 10 min. All chemicals and solvents used were of analytical grade.

### Plant Material

*Centaurea ragusina* L. plants (in vegetative phase) were collected in September, 2016, from two wild habitats – Katalinić brig (43°30′03″N, 16°26′40″E, 363 m) and Sustipan (43°30′04″N, 16°25′35″E, 754 m), Split, Croatia and identified by M. Ruščić, Department of Biology, University of Split, Croatia. A voucher specimen (FSS-CR112016) is deposited at the above-mentioned department.

For extract preparation and isolation of pure compounds, lyophilized leaf materials from both locations were combined after confirmation of their comparable metabolite profile (Vujčić et al., 2017).

### Extraction and Isolation

The dried ground leaves of *C. ragusina* L. (804.9 g) were macerated with 7 L EtOH 96% (at 22°C for 7 days). For an exhaustive extraction the procedure was repeated three times. The dried extract (CRE, 108.9 g) was roughly fractionated by silica gel CC (Merck silica gel 60 PF254, 510 g; 5.5 cm × 56 cm) using a step gradient of CH_2_Cl_2_-EtOAc-MeOH (CH_2_Cl_2_; CH_2_Cl_2_-EtOAc 98:2; 95:5; 90:10; 85:15; 80:20; 75:25; 65:35; 60:40; 55:45; 45:55; 35:65; 25:75; EtOAc; EtOAc-MeOH 80:20; 60:40; 40:60; 20:80; MeOH) to give twelve fractions (A1–12).

Fraction A6 (2.9 g) was further separated using silica gel CC (Merck silica gel 60 PF_254_, 213 g; 3 cm × 56 cm) applying again a gradient system of CH_2_Cl_2_-EtOAc-MeOH to yield 25 fractions (B1–25). Fraction B11 (91.6 mg) was purified via Sephadex LH-20 CC (mobile phase: MeOH) yielding eight fractions (C1–8). Fraction C7 was obtained as 17.5 mg of compound **2** (oroxylin A). Also Fraction B12 (76.4 mg) was purified via Sephadex LH-20 CC (mobile phase: MeOH) yielding 14 fractions (D1–14). Fraction D13 was obtained as 11.8 mg of compound **1** (chrysin).

Fraction B19 (939.8 mg) was subjected to silica gel CC (Merck silica gel 60 PF_254_, 310 g; 3.3 cm× 63 cm) eluting with the isocratic solvent system of *n*-hexane-EtOAc-CH_3_COOH (6:3:1), yielding 14 fractions (E1–14). Fraction E9 (26.9 mg) was further separated by means of a Sephadex LH-20 column (mobile phase: MeOH) to give three fractions (F1–3). Fraction F2 was obtained as 21.8 mg of compound **5** [(3aR,4S,6aR,8S,9aR,9bR)-[dodecahydro-8-dihydroxy-3,6,9-tris(methylene)-2oxo-2(3H)-azuleno[4,5-b]furanyl]-3-methyl-butanoate].

Fraction A8 (834.3 mg) was separated via a Sephadex LH-20 column (mobile phase: MeOH) giving seven fractions (G1-7). Fraction G7 was obtained as 80.3 mg of compound **3** (hispidulin). Fraction G4 (339.4 mg) was submitted to passage over a Sephadex LH-20 column (mobile phase: CH_2_Cl_2_-acetone, 85:15) to yield twelve fractions (H1-12). Fraction H10 (251.4 mg) was further separated using silica gel CC (Merck silica gel 60 PF_254_, 150 g; 1.5 cm × 56 cm) applying again a gradient system of *n*-hexane-EtOAc-CH_3_COOH (6:3:1 to 4:5:1) to yield ten fractions (I1-10). Fraction I9 (185.8 mg) was subjected to a Sephadex LH-20 column (mobile phase: MeOH) resulting in three fractions (J1-3). Fraction J1 (17.8 mg) was purified by silica gel CC (Merck silica gel 60 PF_254_, 50 g; 1.5 cm × 35 cm) applying again a gradient system of CH_2_Cl_2_-EtOAc-MeOH to yield two fractions (K1-2). Fraction K1 was obtained as 3.4 mg of compound **4** (deacylcynaropicrin). Fraction J2 (153.6 mg) was purified by preparative TLC (Merck silica gel 60 PF_254_, 20 cm × 20 cm) and a solvent system of *n*-hexane-EtOAc-CH_3_COOH (5:4:1) to yield three fractions (L1-3). Fraction L2 (115.7 mg) was separated via a Sephadex LH-20 column (mobile phase: MeOH) giving two fractions (M1-2). Fraction M2 was obtained as 12.7 mg of compound **6** (hemistepsin A).

The physical and spectroscopic data of compounds **1** to **6** agreed with those published previously for chrysin, oroxylin A, hispidulin, deacylcynaropicrin, [3aR,4S,6aR,8S,9aR,9bR)-[dodecahydro-8-dihydroxy-3,6,9-tris(methylene)-2oxo-2(3H)-azuleno[4,5-b]furanyl]-3-methyl-butanoate], and hemistepsin A (Miyase et al., 1985; Zdero et al., 1989; Jang et al., 1999; Nagao et al., 2002; Marques et al., 2010; Yang et al., 2013). Their purity was checked using TLC and LC-MS and revealed to be > 98% in all cases.

### Spectroscopic Experiments for the Determination of ctDNA Interactions

The electronic absorption spectra (UV/Vis) were recorded on a Varian Cary 100 Bio spectrophotometer (Agilent, Santa Clara, CA, United Staets) and circular dichroism (CD) spectra on a JASCO J815 spectrophotometer (ABL&E Handels GmbH, Wien, Austria) at 25°C using appropriate 1 cm path quartz cuvettes (Eriksson and Nordén, 2001). The calf thymus DNA (ctDNA) was purchased from Sigma-Aldrich. Isothermal titration calorimetry (ITC) experiments were performed on a MicroCal VP-ITC microcalorimeter (MicroCal, Inc., Northampton, MA, United States) (Chaires, 2006). Origin 7.0 software, supplied by the manufacturer was used for data analysis. All additional data of these experiments are provided in the **Supplementary Materials**.

### Antibacterial Assay

Antibacterial activities of *C. ragusina* L. CRE extract, fractions and isolated compounds against Gram-negative *A. baumannii* Durn (Ćurković-Perica et al., 2015) and Gram-positive *S. aureus* ATCC 25923 were tested using modified Clinical and Laboratory Standards Institute (CLSI), broth microdilution (BD) using 2,3,5-triphenyltetrazolium chloride (TTC) (Lee et al., 2007). The TTC-BD were performed according to the guidelines of the CLSI using 96-well microplates (Clinical Laboratory Standards Institute [CLSI], 2007). The bacteria were grown on nutrient agar (Biolife, Milan, Italy) for 16 h at 36 ± 0.1°C to obtain the cultures in log phase of growth. The bacterial biomass was then suspended in sterile NaCl (0.85% v/v) to give turbidity equivalent to the McFarland 0.5 standard. Bacterial suspension (0.1 mL) was transferred to a tube containing 9.1 mL nutrient broth (Biolife) and 0.8 mL 0.05% TTC to give an inoculum density of 1 × 10^6^ Colony Forming Units (CFU)/mL. Minimum inhibitory concentration (MIC) and minimum bactericidal concentration (MBC) values were determined in triplicates. The final concentrations for MIC and MBC determination of samples were 1.9–4000 μg/mL. Other data on antibacterial experiments are available in the **Supplementary Material**.

### Cytotoxicity Assays and Cell Death Analysis

#### Crystal Violet (CV) Assay

Murine melanoma (B16F10) cell lines, human colon carcinoma (Caco-2) and human breast carcinoma (MCF-7) cell lines were purchased from American Type Culture Collection (ATCC, Manassas, VA, United States), murine fibrosarcoma (FsaR) and murine squamous cell carcinoma (SCCVII) cell lines were obtained from BC Cancer Research Centre (Vancouver, Canada). Cells were grown in a humidified atmosphere of 5% CO_2_, at 37 °C in Roswell Park Memorial Institute (RPMI) 1640 medium supplemented with 10% fetal bovine serum (FBS) (Sigma-Aldrich, St. Louis, MO, United States). As normal cell line, the V79 fibroblasts derived from hamster’s lung tissue, were used. CV protocol (Ivanković et al., 2015) is described in **Supplementary Material**.

#### [3-(4,5-Dimethylthiazol-2-yl)-2,5-Diphenyltetrazolium Bromide] Tetrazolium Reduction (MTT) Assay

Ethacrynic acid (ETA; Sigma-Aldrich) was dissolved in DMSO (Sigma-Aldrich) and kept at -20°C. Buthionine sulfoximine (BSO; Sigma-Aldrich) and N-acetylcysteine (NAC; Sigma-Aldrich) were dissolved in water, 3-(4,5-dimethyl-2-thiazolyl)-2,5-diphenyl-2H-tetrazolium bromide was purchased by Sigma-Aldrich and dissolved in phosphate-buffered saline and kept by 4°C. Human cervical carcinoma HeLa cell line was obtained from cell culture bank (GIBCO BRL-Invitrogen, Waltham, MA, United States). The cells were grown as a monolayer culture in Dulbecco’s modified Eagle’s medium (DMEM; Sigma-Aldrich), supplemented with 10% (FBS; Sigma-Aldrich) in a humidified atmosphere of 5% CO_2_ at 37°C and were sub-cultured every 3–4 days. Cytotoxic activity of the STL **5** was determined by MTT assay, an assay for assessing cell viability based on its metabolic activity, modified accordingly (Mickisch et al., 1990; also in **Supplementary Material**).

#### Cell Cycle and Cell Death Analysis

HeLa cells were seeded into tissue culture plates and treated with different concentrations of the compound during 72 h. Thereafter, both adherent and floating cells were collected, washed with PBS and fixed overnight in 70% ethanol at –20°C. Fixed cells were treated with RNase A (0.1 mg/mL, Sigma-Aldrich) for 1 h at room temperature and afterward stained with propidium iodide (PI; 50 μg/mL, Sigma-Aldrich) for 30 min in the dark. In order to analyze the cell cycle progression, the DNA content and PI staining were detected by flow cytometry (FACS Calibur, BD Biosciences, San Jose, CA, United States). Data were analyzed with ModFit LT^TM^ program (Verity Software House Inc., Topsham, ME, United States).

Twenty-four hours after the seeding, HeLa cells were treated with 2, 5, and 10 μM of compound **5**. After 48 h, both adherent and floating cells were collected by centrifugation and then washed with PBS. The cell suspension was incubated with Annexin V (BD Biosciences; according to producer’s protocol) and PI (5 μg/mL, Sigma-Aldrich). Upon 30 min incubation at room temperature in the dark, the viable, early apoptotic, late apoptotic/necrotic, and necrotic cell populations were detected and counted by flow cytometry (BD Biosciences). Data were analyzed with ModFit LT^TM^ program (Verity Software House Inc.).

In addition, the specific markers of programmed cell death were determined, cleavage of Poly (ADP-ribose) polymerase-1 (PARP) and caspase-3, by western blot as described previously (Brozovic et al., 2013). In short, the 2 h incubation at room temperature with monoclonal anti-PARP (Santa Cruz Biotechnology) and polyclonal anti-caspase-3 (anti-Cas-3; Cell Signaling Technology, Danvers, MA, United States) antibodies was performed. After washing with 0.01% Tween 20 in PBS and incubation with the corresponding horseradish peroxidase-coupled secondary antibody (Amersham Pharmacia Biotech, Munich, Germany), proteins were visualized with ECL (Amersham Pharmacia Biotech) according to the manufacturer’s protocol. All membranes were incubated with anti-extracellular-signal-regulated kinases 1/2 (anti-ERK1/2) (Santa Cruz Biotechnology) antibody to confirm equal protein loading. ERK1/2 was used as loading controls since no changes in total ERK1/2 expression were detected upon exposure of cells to different drugs (Brozovic et al., 2004; Herraiz et al., 2011).

#### Determination of Glutathione Function

The function of intracellular GSH in cell response to STL **5** was investigated by MTT assay. HeLa cells were either pre-treated overnight with a specific inhibitor, 0.001 mM BSO or for 2 h with a precursor in GSH synthesis, 5 mM NAC. Both compounds are frequently used in the manipulation of GSH level in the cells (Trachootham et al., 2009; Kannan et al., 2014). Upon pre-treatment with either BSO or NAC, different concentrations of STL **5** were added and the cytotoxicity effect of the compounds was determined 72 h later as described above.

The capacity of GSH to form the detoxification conjugates through enzymatic reaction with STL **5** was investigated by pretreatment of HeLa cells with of 5 μg/mL ETA for 2 h and then with different concentrations of STL **5**. The cell survival was examined 72 h after. The optimal concentrations of used modulators of GSH synthesis and glutathione S-transferase reaction were determined previously (Osmak and Eljuga, 1993; Brozovic et al., 2008; Brozovic et al., 2013).

### Statistical Analysis

All results were evaluated using the software package Statistica 12.0 (StatSoft, Tulsa, OK, United States). Results were subjected to one-way ANOVA for comparison of means and significant differences were calculated according to Duncan’s multiple range test. Data were considered statistically significant at *P* < 0.05. Different letters indicate significant difference at *P* < 0.05.

## Results

### Selection of Plant Material, Extraction, Fractionation, and First Bioactivity Assessment

**FIGURE 1:**
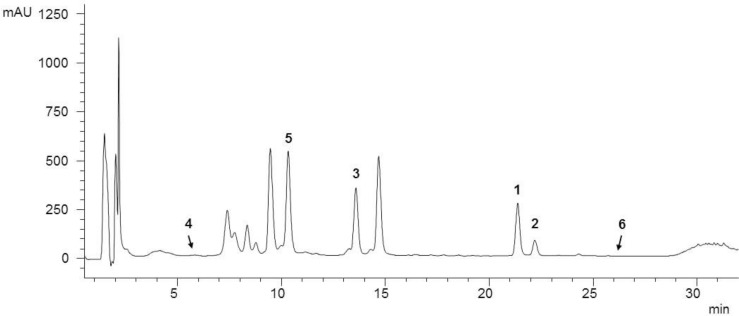
HPLC analysis of the ethanol *C. ragusina* L. leaf extract (CRE) at 254 nm (for LC parameters see section “Experimental”).

As a starting point for phytochemical investigations, an ethanol *C. ragusina* L. leaf extract (CRE) was prepared to obtain a multicomponent mixture embracing a wide range of secondary metabolites (**Figure 1**).

In antibacterial assays, CRE showed moderate antibacterial effects against *S. aureus* with MIC of 500 μg/mL and MBC of 2000 μg/mL, respectively, and weak activity against *A. baumannii* with MIC and MBC values both of > 4000 μg/mL, respectively (Vujčić et al., 2017).

In contrast to the moderate antibacterial activity, CRE exhibited significant effects on all tested murine melanoma (B16F10), squamous cell carcinoma (SCCVII) fibrosarcoma (FsaR) cell lines and normal hamster fibroblasts (V79).

At a CRE concentration of 60 μg/mL, the cell survival relative to the negative control was ≤ 10% for V79 fibroblasts, SCCVII and FsaR, respectively, and 21% for the B16F10. CRE did not show selective cytotoxicity between murine cancer and normal cell lines, which may be a consequence of a cumulative effect of different bioactive compounds included in the crude extract.

For a more focused isolation process of the bioactive constituents of the crude extract, twelve fractions (A1–A12) obtained by separation of CRE via silica gel chromatography were retested in the mentioned cell lines, revealing A6 and A8 as fractions with the strongest cytotoxic activity. Both fractions (60 μg/mL) exhibited stronger cytotoxic activity on B16F10 (< 10% cell survival) and SCCVII (≤ 20% cell survival) cell lines compared to the moderate activity observed toward FsaR and V79 cells (≤ 30% cell survival). Based on these results, fractions A6 and A8, were selected for further chromatographic separations.

### Isolation and Identification of Pure Compounds

Six constituents were isolated from CRE fractions A6 and A8 by separation techniques including CC and preparative thin layer chromatography.

**FIGURE 2:**
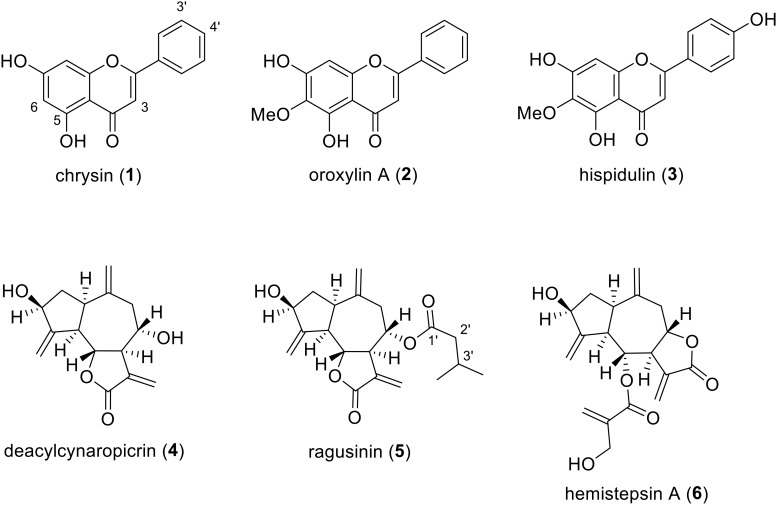
Chemical structures of isolated *C. ragusina* L. leaf constituents.

By using HR-ESI-MS analyses and NMR experiments, and by comparison with earlier studies (Miyase et al., 1985; Zdero et al., 1989; Jang et al., 1999; Nagao et al., 2002; Marques et al., 2010; Yang et al., 2013), the isolates (**Figure 2**) were identified as chrysin (**1**), oroxylin A (**2**), hispidulin (**3**), deacylcynaropicrin (**4**), (3aR,4S,6aR,8S,9aR,9bR)-[dodecahydro-8-dihydroxy-3,6,9-tris(methylene)-2oxo-2(3H)-azuleno[4,5-b]furanyl]-3-methyl-butanoate (**5**), and hemistepsin A (**6**). Instead of using the complicated and long systematic name for compound **5**, we created the trivial name ragusinin. The flavonoids (**1–3**) can be classified as flavones, whereby different substitution patterns with methoxy or hydroxy groups can be observed on C-6 and C-4′. Compounds **4**–**6** are STLs belonging to the subtype of guajanolides.

Compound **1** is a frequently occurring flavone reported as a constituent of several *Centaurea* species (Mouffok et al., 2012). However, we are reporting here for the first time its isolation from *C. ragusina* L. Different bioactivities have been reported for compound **1**. It suppresses inducible nitric oxide synthase, cyclooxygenase-2 expression and inhibits NF-κB activation, which altogether leads to anti-inflammatory effects (Feng et al., 2014). Compound **1** is also reported as a tumor cell growth arrest compound, arresting C6 glioma cells in the G1 phase of the cell cycle either through activating p38-MAPK leading to the accumulation of p21Waf1/Cip1 protein or mediating the inhibition of proteasome activity (Weng et al., 2005). It also suppresses tumor growth of anaplastic thyroid cancer ATC cells both *in vitro* and *in vivo* (Yu et al., 2013).

Compound **2** is known as the main component of several medicinal plants including *Oroxylum indicum* (Krueger and Ganzera, 2012) and various *Centaurea* species. However, so far it has not been reported for *C. ragusina* L. Compound **2** was shown to activate caspase-3 and caspase-9 in human colon carcinoma HCT-116 cells and decrease tumor volume and weight in immunodeficient mice that were inoculated with HCT-116 cells (Hu et al., 2012). It also exhibits anti-inflammatory effects by decreasing pro-inflammatory cytokines mediated by estrogen receptor activity (Wang et al., 2013).

Compound **3** has been isolated from various *Centaurea* species, e.g., *C. melitensis* L. (Negrete et al., 1989), *C. aspera* L. (Ferreres et al., 1980), and *C. jacea* L. (Forgo et al., 2012), but never from *C. ragusina* L. It is an important compound used in traditional Chinese medicine for the treatment of liver carcinoma (Gao et al., 2014). Besides an apoptotic effect on human liver cancer HepG2 cells, it was also shown that this effect is mediated via mitochondrial dysfunction (Gao et al., 2014). Furthermore, an anti-proliferative effect toward human lung cancer A-549 cells was reported for compound **3** (Zhang et al., 2012).

Compound **4** has already been reported as a constituent of *C. ragusina* L. (Mahmoud et al., 1986) and other *Centaurea* species with anti-inflammatory and cytotoxic activity (González et al., 1977; Sosa et al., 2011).

To the best of our knowledge, guajanolides **5** and **6** have never been identified as constituents from *Centaurea* species before. Whereas for **6** an antibacterial and cytotoxic activity toward human cell lines in the low μM range has previously been reported (Jang et al., 1999) compound **5** (ragusinin) with its isovalerate residue is a rare STL without reports on bioactivity.

### Study of Biological Activity (Interactions With DNA, Antibacterial and Cytotoxic Activity) of *C. ragusina* L. Constituents

The DNA binding affinity of compounds is important to explore since the DNA represents a well-known target of several broadly used drugs and the binding to DNA is one of the common causes of cell death (Demeunynck et al., 2002; Sangeetha Gowda et al., 2014). In order to determine the binding affinity of the isolated compounds to ctDNA, UV/Vis spectroscopy and ITC (Bronowska, 2011) were employed (see section “Experimental” and “**Supplementary Material** for details”). CD was used for monitoring of conformational changes of ctDNA induced by small molecule binding and for gaining information about modes of interaction (Cantor and Schimmel, 1980; Johnson, 1994).

Among isolated STLs and flavonoids (**Supplementary Material**), only **5** exhibited significant changes in CD titrations (**Supplementary Figure S4**). Due to that reason, we decided to characterize the binding of DNA only with compound **5**. UV/Vis spectroscopy was not applicable in the study of DNA-ragusinin interaction due to absorption of STLs at short wavelengths (210–220 nm). Therefore, the binding interaction of **5** with ctDNA was monitored by ITC.

**FIGURE 3:**
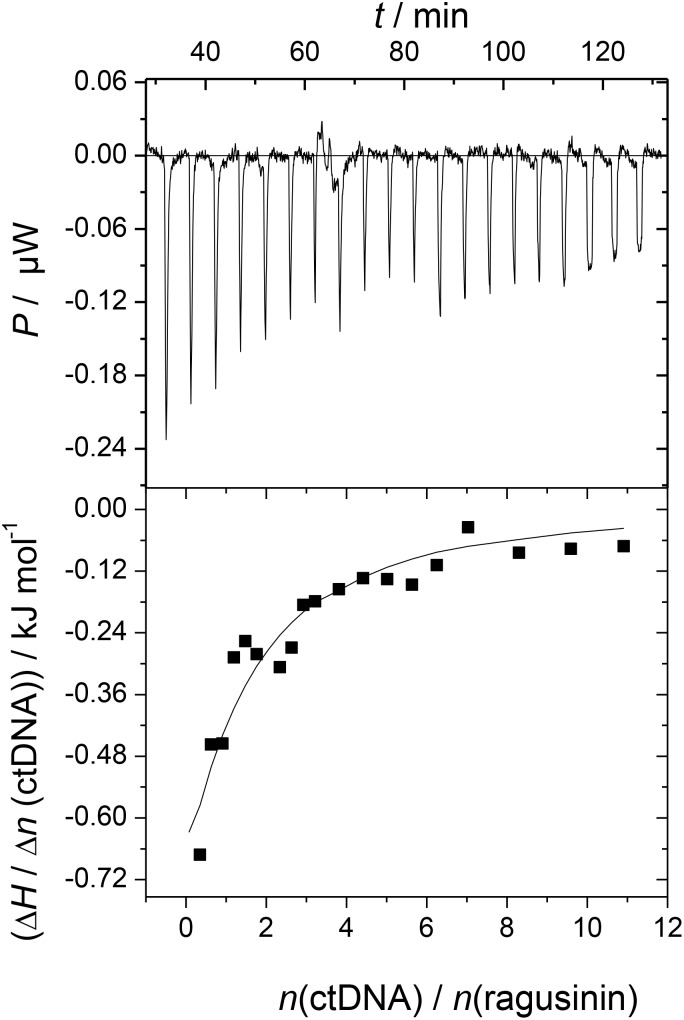
Calorimetric titration of compound **5** (*c* = 3 × 10^-5^ mol dm^-3^) with ctDNA (*c* = 1.5 × 10^-3^ mol dm^-3^) in sodium cacodylate buffer (pH 7.0, *I* = 0.05 mol dm^-3^; *T* = 25°C; Δ_r_*G*/kJ mol^-1^ = -23.0). The top panels represent the raw data from the single injection of ctDNA into a solution of **5** and the bottom panels show the experimental injection heats while the solid lines represent the calculated fit of the data.

The ITC experiment of **5** with ctDNA resulted in negative peaks indicating that the binding process was exothermic (**Figure 3**). The resulting values were fitted to a single-site binding model by the non-linear least square method yielding rather moderate binding constant (log *K*_a_ = 4.04). The stoichiometry (N) was fixed to 0.5 based on results from CD titration with ctDNA (the saturation of binding sites was reached at the ratio, *r* = 0.5, **Supplementary Material**). The binding of **5** to ctDNA was characterized by a positive binding entropy (*T*Δ_r_*S*/kJ mol^-1^ = 18.5) accompanied by smaller negative enthalpy (Δ_r_*H*/kJ mol^-1^ = -4.6) revealing that its binding is entropically driven. In many cases, the groove binding is associated with positive (favorable) binding entropies due to the release of confined or interfacial water molecules to the bulk (Perozzo et al., 2004; Chaires, 2006).

Two STLs **5** and **6** were found to be the most active ones reducing the growth of *S. aureus* with a MIC value of 31.3 μg/mL (**Supplementary Table S1**). However, since the cytotoxic activity of the isolated compounds was more prominent than the antibacterial, we presented here cytotoxic activity in more detail while the data on antibacterial activity are available in the **Supplementary Material**.

**FIGURE 4:**
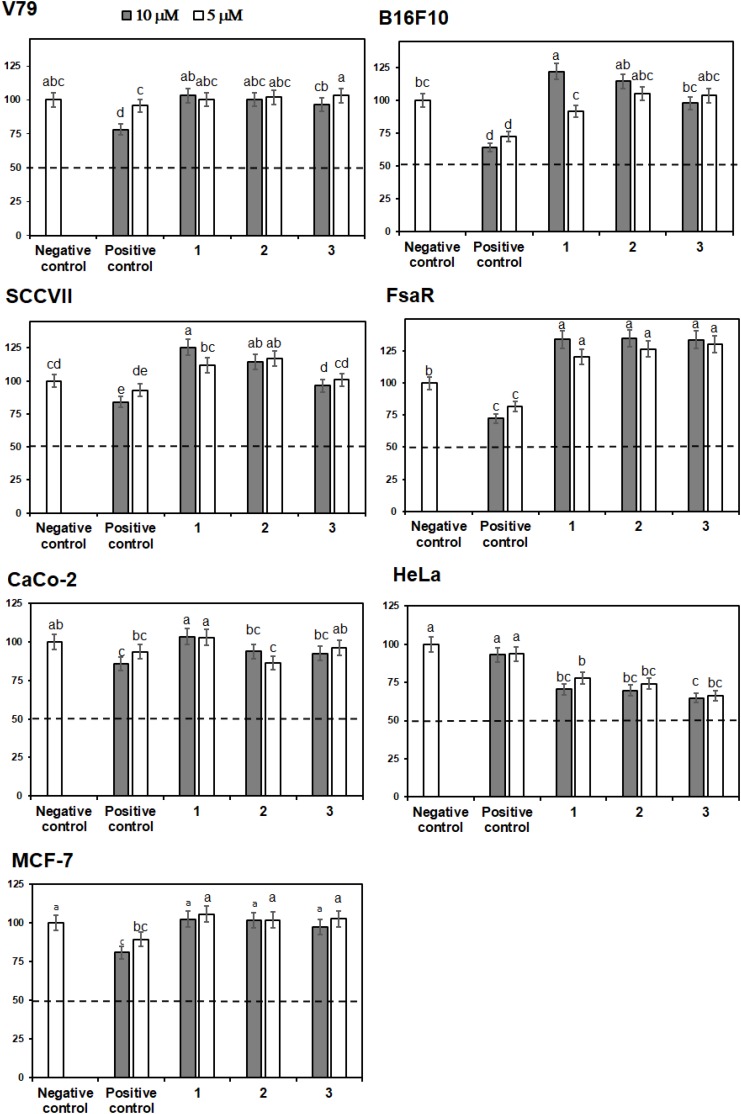
Percentage of cell survival of rodent (V79, SCVII, B16F10, and FsaR) and human (Caco-2, HeLa, and MCF-7) cell lines after exposure to isolated flavonoids (compounds **1**–**3**), at concentrations of 10 μM (gray bars) and 5 μM (white bars). Values represent mean of 3 replicates ± SD. Different letters indicate significant difference at *p* < 0.05. The dashed line indicates inhibition of cell growth by 50%. The positive control is 5-fluorouracil and the negative control are cells without the tested samples.

**FIGURE 5:**
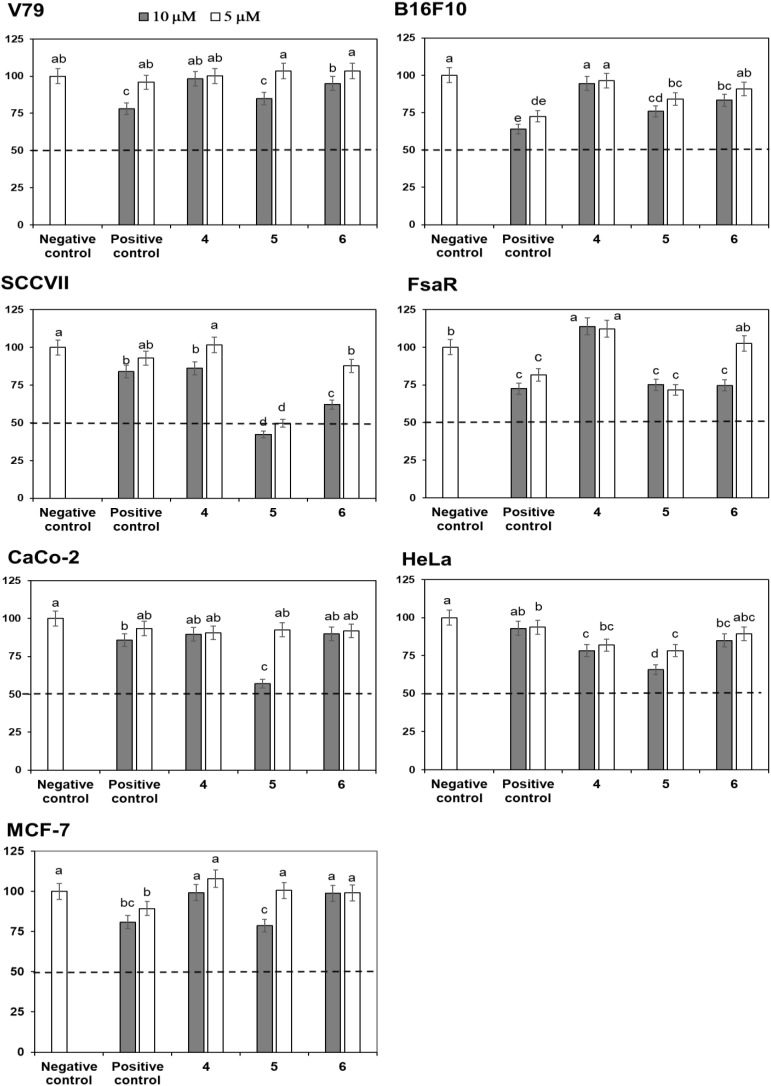
Percentage of cell survival of murine (V79, SCVII, B16F10, and FsaR) and human (Caco-2, HeLa, and MCF-7) cell lines after exposure to deacylcynaropicrin (**4**), ragusinin (**5**) and hemistepsin A (**6**) at concentrations of 10 μM (gray bars) and 5 μM (white bars). Values represent means of three replicates ± SD. Different letters indicate significant difference at *p* < 0.05. The dashed line indicates inhibition of cell growth by 50%. The positive control is 5-fluorouracil and the negative control are cells without the tested samples.

Crystal Violet (CV) bioassay, which measures the DNA mass of living cells, was used for initial activity screening. The cytotoxic effect of the isolated compounds **1** to **6** was monitored with the CV test for 24 h at 5 and 10 μM on a panel of three murine and three human cancer cell lines. 5-Fluorouracil was used as a positive control at equimolar concentrations as the studied compounds (**Figures 4**, **5**). The isolated flavonoids **1** and **2** did not demonstrate cytotoxic activity against the majority of cancer cell lines at the applied concentrations. Only compound **3** exerted a noticeable cytotoxic effect against HeLa cells (**Figure 4**). Among the isolated STLs, **5** showed the most prominent cytotoxic activity. In particular, compound **5** reduced the cell survival of SCCVII cells to 42% at 10 μM and 50% at 5 μM concentration (**Figure 5**). It also exhibited promising activity against Caco-2 cells at 10 μM and HeLa cells at both concentrations applied (**Figure 5**). On the other hand, **5** had weak (cell survival was 85% at 10 μM) or no activity (> 95% at 5 μM) against normal fibroblasts (V79). Moreover, while compound **4** did not show significant cytotoxic effects on any cell line at the applied concentrations, compound **6** showed noticeable effects against SCVII and FsaR cells at 10 μM.

### Cytotoxic Activity of Ragusinin (**5**) in HeLa Cell Line

In order to determine the biological effect of the most active compound **5** (**Figure 5**) in more detail, we used MTT assay and HeLa cells as experimental model (Cimbora-Zovko et al., 2011).

**FIGURE 6:**
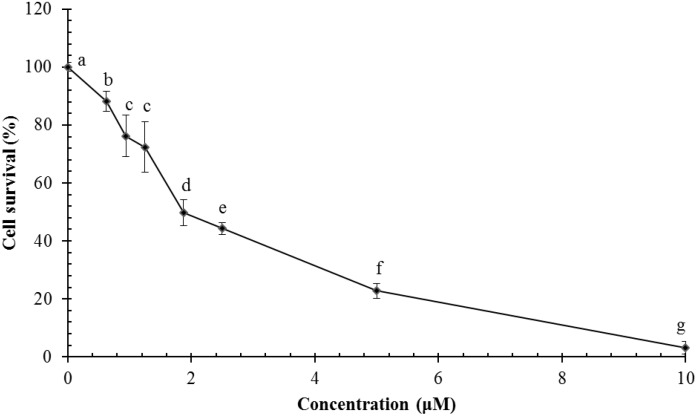
Survival of HeLa cells following treatment with ragusinin (**5**). The cells were treated for 72 h with different doses of **5**. The MTT assay was performed. Each point represents the mean of quadruplicates ± SD. Different letters indicate significant difference at *p* < 0.05. The experiment is performed at least three times.

Ragusinin decreased cell survival of HeLa cells in a concentration-dependent manner in comparison to untreated cells (**Figure 6**). The dose that killed 50% of the cell population (IC_50_ value) after 72 h of continuous treatment with **5** was between 1.8 and 2.3 μM (**Figure 6**).

**FIGURE 7:**
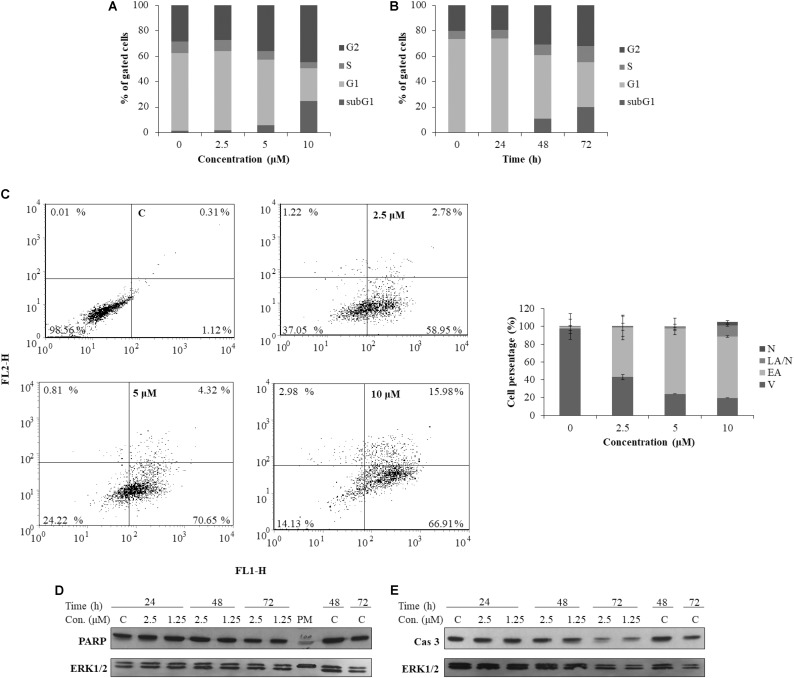
Cell cycle progression and induction of cell death in HeLa cells following treatment with ragusinin (**5**). The cells were treated either with 2.5, 5, and 10 μM of compound **5** for 48 h **(A)** or with 10 μM during 24–72 h **(B)**. Cell cycle analysis was performed by flow cytometry upon cell staining with PI. The representative data of three independent experiments which yielded similar results are shown. The cells were either non-treated or treated with 2.5, 5, and 10 μM of **5**. After 48 h and staining with Annexin V and PI, the cell death was measured by flow cytometry. The representative data of three independent experiments which yielded similar results are shown. The statistical analysis of data for three independent experiments are presented as means of percentage of viable (V), early apoptotic (EA), late apoptotic/necrotic (LA/N), and necrotic (N) cell populations ± standard deviation **(C)**. 24–72 h after exposure to 1.25 and 2.5 μM of **5**, protein level of cleaved PARP **(D)** and activated (i.e., cleaved) caspase-3 (Cas 3) **(E)** and cleaved PARP was analyzed in whole cell extracts by Western blot analysis. ERK1/2 protein expression was used as internal protein loading control. The representative data of three independent experiments which yielded similar results are shown. Zero or C, non-treated cells; PM, protein marker PageRuler Prestained Protein Ladder (Thermo Fisher Scientific, United States). FL1-H/Annexin V-FITC, FL2-H/Propidium iodide.

To better understand the mechanism underlying the cell growth impairment by **5**, the cell cycle progression was investigated. HeLa cells were treated with increasing doses of **5** during 48 h. As shown in **Figure 7A**, compound **5** triggers the accumulation of HeLa cells in the G2 phase of cell cycle. Moreover, a compound **5** induced dose-dependent increase of cells in the Sub G1 population indicates a ragusinin-triggered cell death (**Figure 7A**). The same was confirmed by treatment of cells with 10 μM of **5** during 24–72 h. Time-dependent accumulation of HeLa cells in the G2 phase is detectable as well as time-increase of cells in the Sub G1 phase of the cell cycle (**Figure 7B**).

In order to determine the type of cell death triggered by **5**, the cells were treated with increasing doses of the compound and 48 h later, FACS-Annexin V/PI staining was performed. Our results show a ragusinin-induced dose-dependent apoptosis (**Figure 7C**). We then performed Western blot analysis of specific cell death markers, PARP and caspase-3 cleavage, following treatment with 1.25 and 2.5 μM of **5** for 24–72 h. The obtained results were interesting since **5** did not induce PARP (**Figure 7D**) and caspase-3 cleavage (**Figure 7E**). The assumed caspase-independent cell death triggered by **5** was in addition confirmed by measuring the absence of caspase activity 3/7 by Caspase-Glo^®^ 3/7 Assay (data not shown). The occurrence of caspase and PARP cleavage independent cell death indicates some alternative cell death pathway described in the literature to be triggered by a different type of cell stressors (Kroemer and Martin, 2005; Tait and Green, 2008). This is the first example of caspase independent cell death described for compound **5**.

### Role of Glutathione (GSH) in Protection of Cells From Ragusinin (**5**)

**FIGURE 8:**
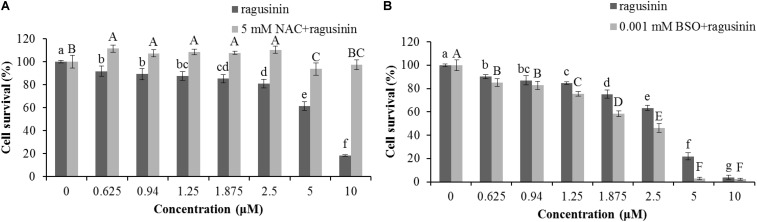
The role of GSH in ragusinin’s (**5**) toxicity. The cells were either pre-treated for 2 h with 5 mM NAC **(A)** or overnight with 0.001 mM BSO **(B)**. After that, the cells were treated with different concentrations of **5**. 72 h later, an MTT assay was performed. Bars represent the mean of quadruplicates ± SD. Different letters indicate significant difference at *p* < 0.05. All experiments were performed at least three times.

We were further interested in the possible role of GSH as a protector of cells in ragusinin-induced cell death. For that purpose, HeLa cells were either pre-treated with 5 mM NAC, the precursor for GSH synthesis, for 2 h prior to treatment with compound **5** or overnight with a specific inhibitor of GSH synthesis, i.e., 0.001 mM BSO. The conditions used were tested previously to be effective (Brozovic et al., 2008; Brozovic et al., 2014). The obtained data showed that an increased level of GSH protects HeLa cells from ragusinin’s toxicity (**Figure 8A**). At the same time, depletion of GSH decreased cell survival of HeLa cells compared to cells treated with compound **5** only (**Figure 8B**).

**FIGURE 9:**
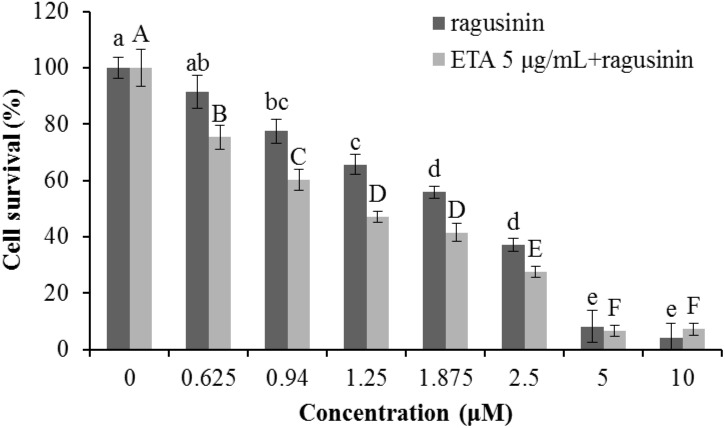
The treatment of HeLa cells with ethacrynic acid (ETA). The cells were pre-treated with 5 μg/mL ETA. Two hours later the cells were treated with different concentrations of **5**. 72 h later an MTT assay was performed. Bars represent the mean of quadruplicates ± SD. Different letters indicate significant difference at *p* < 0.05. All experiments were performed at least three times.

Due to the fact that GSH instills several vital roles within a cell including antioxidation, maintenance of the redox state, modulation of the immune response, and detoxification of xenobiotics (Balendiran et al., 2004), we discussed first its possible role as an antioxidant. It is known that the cytotoxicity of the sesquiterpenes helenalin and cynaropicrin can be affected via generation of intracellular reactive oxygen species (ROS) (Cho et al., 2004; Jang et al., 2013). We examined that possibility for ragusinin by pre-treating HeLa cells either with tempol or trolox, two antioxidant compounds (Aliaga et al., 2003). Results showed that antioxidants, as well as salubrinal (Brozovic et al., 2013), i.e., an inhibitor of endoplasmic reticulum stress, had no impact on ragusinin’s toxicity (**Supplementary Material**, in **Supplementary Figure S6**). Thus, we further explored the capability of **5** to reach functional protein targets in living cells without being deactivated by reaction with GSH. The combination treatment of HeLa cells with the well-known inhibitor of glutathione S-transferase (GST) ethacrynic acid (Osmak et al., 1998) (*c* = 5 μg/mL) decreased cell survival compared to cells treated with **5** only (**Figure 9**). The data imply the enzymatically regulated formation of a detoxification complex between GSH and **5**.

## Discussion

Among the isolates (**1** to **6**) from the traditionally used herbal remedy *C. ragusina* L. leaves, we discovered an interesting pharmacological profile for the rare guajanolides ragusinin (**5**) and hemistepsin A (**6**). Compound **5** was only once described as a constituent from the aerial parts of the Australian *Helipterum maryonii* S. Moore (Zdero et al., 1989) and is characterized by an isovalerate residue in position C-9a; compound **6** has been described previously as a constituent from leaves and flowers of *Hemisteptia lyrata* Bunge (Jang et al., 1999).

Whereas both natural compounds showed no antibacterial activity against the Gram-negative *A. baumannii*, they exhibited moderate inhibitory activity against *S. aureus* ATTC 25923 (MIC value of 31.3 μg/mL, **Supplementary Material**). For comparison, three STLs (13-acetylsolstitialin A, centaurepensin, and chlorojanerin) isolated from the aerial parts of *Centaurea solstitialis* L. ssp. *solstitialis* showed inhibitory activity against standard *S. aureus* with MIC values of 16 μg/mL (Özçelik et al., 2009). In line with these findings, we focused on a more prominent cytotoxic activity of the isolated compounds and the investigation of the mechanism of action of the most active compound **5**.

We investigated the binding of isolated compounds to DNA since studying the interactions of compounds with potential drug targets and the knowledge of their antiproliferative activity can help in forming the hypothesis about the mechanism of action of novel compounds.

The isolated flavonoids (**1–3**) exhibited only weak interactions with DNA which may explain the absence of cytotoxic effects against the majority of studied cell lines. The somewhat stronger cytotoxic activity of flavonoids against HeLa cells can be ascribed to interactions with biological targets other than DNA (Chen et al., 2012). Compound **3** with the highest number of hydroxy groups on the flavonoid skeleton exhibited the most pronounced cytotoxic activity. This finding is in agreement with literature data (Beutler et al., 1998; Jeong et al., 2007; Csupor-Löffler et al., 2009).

While there are numerous reports on DNA binding affinities with flavonoids (Kanakis et al., 2005; Rusak et al., 2010), there is little data on DNA binding interactions with sesquiterpenes (Vujčić et al., 2007). Regarding the structure of **5** and literature data (Gates, 2009; Chadwick et al., 2013), the interaction with DNA can be achieved via noncovalent binding to double-stranded DNA (dsDNA) or by alkylation of DNA nucleophiles through reaction with the α-methylene-γ-lactone group. Several studies on the reactivity of STLs toward OH- or N-nucleophiles (Atta-ur-Rahman, 2011) and CD changes which were not consistent with an alkylation effect (Agarwal et al., 2014) (**Supplementary Figure S4**) do not support the latter possibility. In addition, the -SH group was found to be much more susceptible to alkylation by sesquiterpenes than other nucleophiles (Gewirtz et al., 2007). A reasonable explanation based on our results from ITC and CD is noncovalent binding of **5** most probably inside a hydrophobic interior of the DNA groove. Due to its rather moderate binding affinity to ctDNA, it can be concluded that the cellular DNA is not **5’**s primary target in the living cell.

Among the isolated STLs, compound **5** showed the strongest cytotoxic activity, especially on murine SCVII as well as human Caco-2 and HeLa cell lines, while no cytotoxic effect on normal V79 fibroblasts was observed. Similar to the antibacterial activity, a correlation between the cytotoxic activity and the type and properties of the substituents on the central ring in the vicinity of the α-methylene-γ-lactone group was observed. In comparison to less lipophilic substituents of sesquiterpenes, as observed for **6** and especially **4**, the hydrophobic character of the isovalerate residue of **5** most probably enables better penetration through the cell membrane and consequently better antibacterial and cytotoxic activity. In the literature, cytotoxic effects of various STLs have been explained by selective alkylation of growth regulatory macromolecules such as enzymes which control cell division and thus a variety of cellular functions (Lyss et al., 1998; Rasul et al., 2012).

Since compound **5** (ragusinin) showed the strongest activity compared to other isolates and since the biological activity of **5** was not described before we decided to investigate the mechanism of its toxicity in more details. The IC_50_ value of ragusinin was between 1.8 and 2.3 μM (**Figure 6**). This is consistent with data from the literature, where compounds of similar chemical structure, i.e., sesquiterpene lactones such as helenalin (Gertsch et al., 2003), neoambrosin (Saeed et al., 2015), and damsin (Villagomez et al., 2013), are reported to be cytotoxic against various cell lines in the micromolar range. Further analysis revealed time-dependent accumulation of HeLa cells upon ragusinin treatment in the G2 phase and Sub G1 phase of the cell cycle (**Figure 7B**). But, the occurrence of caspase and PARP cleavage independent cell death indicates some alternative cell death pathway described in the literature to be triggered by a different type of cell stressors (Kroemer and Martin, 2005; Tait and Green, 2008). The phenomenon is interesting to follow up further, especially because it is known that helenalin, a STL isolated from *Arnica montana*, induces the same atypical form of cell death which does not include the activation of classical mediators of apoptosis (caspases, AIF, Omi/HtrA2, and Apaf/apoptosome) (Hoffmann et al., 2011).

In a series of noteworthy reports on the reactivity and kinetics of helenalin and helenanolide type STLs with GSH, it was demonstrated that at physiological pH helenalin reacts with GSH almost exclusively via its cyclopentenone structure while the α-methylene-γ-lactone site is less reactive (Schmidt et al., 1999). Since the guajanolide **5** possesses one reactive site, i.e., the α-methylene-γ-lactone group that can react with GSH, we decided to investigate the role of GSH in deactivation of **5**.

From the literature is known that GSH is one of the major endogenous antioxidants. In the cytoplasm, GSH is used as a substrate for glutathione peroxidase in the reduction of H_2_O_2_ and lipid hydroxyperoxides, a reaction that produces glutathione disulfide, the so-called oxidative form of GSH. Glutathione disulfide is rapidly reduced to GSH by glutathione reductase. This redox cycling of GSH plays a role in the maintenance of cellular redox homeostasis. GSH binds to endogenous and diverse xenobiotic electrophilic compounds either catalytically, through the action of glutathione S-transferase, or non-catalytically (Townsend and Tew, 2003; Brozovic et al., 2014). The formed GSH conjugates can be exported from cells, resulting in the loss of cellular GSH. Our data showed that variation of GSH amount in the cell induced by a specific precursor in GSH synthesis, NAC or a specific inhibitor of GSH synthesis, BSO had an impact on HeLa survival upon treatment with **5** (**Figure 8**). Similar results were obtained in human colon tumor cells upon BSO and helenalin treatment (Jordan et al., 1987). Moreover, we showed that GSH probably does not play a role as antioxidant (**Supplementary Figure S6**) but rather as molecule which form conjugates with **5** increasing in that way the survival of HeLa cells upon treatment with it (**Figure 9**).

## Conclusion

In this study, the most active compound has shown to be a sesquiterpene lactone, ragusinin (compound **5**) whose biological activity has not been investigated so far. Currently, our knowledge about the mechanism of action of STLs is still limited. Several of them reached clinical trials due to their ability to trigger cell death in tumor but not in normal cells (Zhou and Zhang, 2008; Kawasaki et al., 2009; Crespo-Ortiz and Wei, 2012).

Here we are showing for the first time the biological activity of compound **5**, ragusinin. Although ITC and CD results suggested that the cellular DNA is not the primary target of ragusinin in the living cell, these data represent valuable information since, to the best of found knowledge, STL - DNA interactions have not been communicated before.

The variation of the amount of GSH in the cell by using a specific precursor in GSH synthesis (NAC) or a specific inhibitor of GSH synthesis (BSO) showed the importance of GSH in the cell’s response to **5**. Moreover, it was shown that formation of GSH-ragusinin conjugates increased cell survival what implies the role in GSH detoxification rather than in stabilization of the cell redox system. Ragusinin induced G2 arrest followed by caspase-independent cell death.

Though the actual protein targets remain unclear at this stage of the investigation, we can confirm that compound **5** is deactivated by GSH resulting in a diminished cytotoxic effect.

**FIGURE 10:**
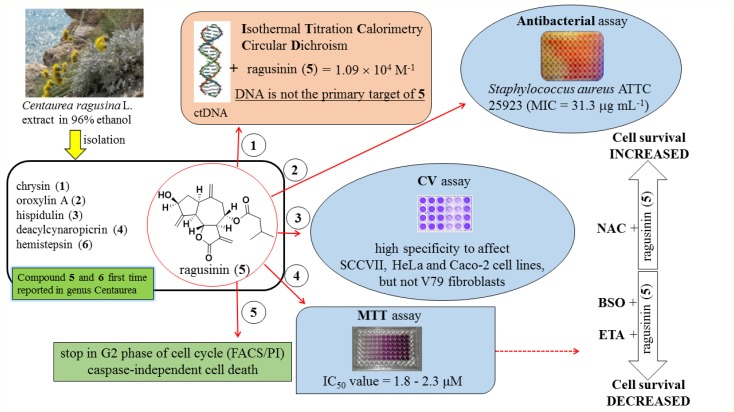
A summary of the most significant results obtained in this study.

A summary of the most significant results obtained in this study by using specified techniques is presented in **Figure 10**. In future studies, it will be interesting to investigate whether ragusinin-GSH conjugates have a biological activity or whether they are ejected from cells through so-called GSH pumps such as MRP1 and MRP2 (Homolya et al., 2003).

## Author Contributions

UG and JR isolated and characterized compounds, analyzed the data, and wrote a part of the manuscript. SRB and VV performed antibacterial assays and processed the data. EU helped in NMR measurements and structure elucidation. RS and SI performed antiproliferative assay by CV method and processed the data. JK and AB performed antiproliferative assay by MTT method, flow cytometry and tests with GSH. AB designed part of the study, analyzed the data, and wrote a part of the manuscript. MRS designed the study, performed DNA binding study (UV/Vis, CD, and ITC titrations), analyzed the data, and wrote a part of the manuscript. All authors participated in the critical reading of the manuscript.

## Conflict of Interest Statement

The authors declare that the research was conducted in the absence of any commercial or financial relationships that could be construed as a potential conflict of interest.
