# Supplementary material for: Biological Activity of Flavonoids and Rare Sesquiterpene Lactones Isolated From Centaurea ragusina L
Source: Front Pharmacol. 2018 Aug 22;9:972. doi: 10.3389/fphar.2018.00972 (PMC6117149; doi:10.3389/fphar.2018.00972)
Supplement: Supplementary file 1 [file Table_1.DOCX]

Supplementary Material

Biological activity of flavonoids and rare sesquiterpene lactones isolated from *Centaurea ragusina* L.

Ulrike Grienke^1^, Sandra Radić Brkanac^2^, Valerija Vujčić^2^, Ernst Urban^3^, Siniša Ivanković^4^, Ranko Stojković^4^, Judith M. Rollinger^1^, Juran Kralj^5^, Anamaria Brozovic^5^*, Marijana Radić Stojković^6^*

*** Correspondence:**PhD Marijana Radić Stojković, Division of Organic Chemistry and Biochemistry, Ruđer Bošković Institute, Zagreb, Croatia, email: [mradic@irb.hr](mailto:mradic@irb.hr)

PhD Anamaria Brozovic, Division of Molecular Biology, Ruđer Bošković Institute, Zagreb, Croatia, email: brozovic@irb.hr

Contents

1. Antibacterial activity

2. UV/Vis experiments and CD experiments

3. ITC experiments

4. Cytotoxic activity

5. Experiments with antioxidants

1. Antibacterial activity of *C. ragusina* L. constituents

*Material and methods*

For the experiments, 50 µL of sample was added into 50 µL of bacterial suspension. The negative control contained 50 µL of bacterial suspension and 50 µL of sterile DMSO (5%). In all the experiments the DMSO concentration in the media did not exceed 2.5%. Experiments were set in triplicate. In all microplate wells, inoculum density for extract/compounds MIC and MBC determination was 5 x 10^5^ CFU mL^–1^. Broth microdilution was performed according to CLSI.

After the incubation in the dark during 24 h at 37 ± 0.1 °C, multiwell plates were shaken and 0.1 mL of sample was plated (spread plate method) directly onto the nutrient agar while another sample was serially diluted (10^–1^ to 10^–8^) and inoculated onto nutrient agar plates. The inoculated plates were incubated at 37 ± 0.1 °C for 24 h. After the incubation period, the bacterial colonies were counted and the number of viable cells was reported as CFU/mL. The numbers of CFU were logarithmically transformed. The antibacterial activity of the *C. ragusina* L. samples was expressed as the percentage reduction of log CFU compared to the corresponding control. Minimum inhibitory concentration (MIC) and minimum bactericidal concentration (MBC) values were determined in triplicates as well. The final concentrations for MIC and MBC determination of samples were 1.9-4000 μg/mL.

*Results of antibacterial activity*

At 62.5 µg/mL, all six isolated compounds induced a reduction of *S. aureus* growth (Table 1). The two sesquiterpene lactones **5** and **6** were found to be the most active ones reducing the growth of *S. aureus* with a MIC value of 31.3 µg/mL (Table 1).

The activity of these sesquiterpene lactones against *S. aureus* may involve a membrane disruption or the inhibition of the activity of the bacterial enzyme MurA (Facey et al., 2010; Ivanescu et al., 2015).

**Supplementary Table 1.** Reduction *S. aureus* (in percent)*,* MIC and MBC after treatment with isolated pure compounds from *C. ragusina* L. compared to the negative control.

| *Staphylococcus aureus ATTC 25923* | | | | | |
| --- | --- | --- | --- | --- | --- |
| compound | % reduction measured at (µg/mL) | | | MIC (µg/mL) | MBC (µg/mL) |
|  | 62.5 | 31.3 | 15.6 |  |  |
| 1 | 28.6 | x | x | 62.5 | > 62.5 |
| 2 | 30.6 | x | x | 62.5 | > 62.5 |
| 3 | 34.9 | x | x | 62.5 | > 62.5 |
| 4 | 42.2 | x | x | > 62.5 | > 62.5 |
| 5 | 68.7 | 31.5 | 28.4 | 31.3 | > 62.5 |
| 6 | 55.5 | x | x | 31.3 | > 62.5 |
| Values represent means of 3 replicates. SD < 5%; x – not measured. Ampicillin MIC <0.12 µg/mL. Negative control is given in Experimental. | | | | | |

The sesquiterpene lactone **4** and the isolated flavonoids (**1**-**3**) were much less active. A correlation was observed in the series of the sesquiterpene lactones between the activity against *S. aureus* and lipophilicity of the substituent in the vicinity of the α-methylene-γ-lactone group. Thus, sesquiterpenes **5** and **6**, with less polar substituents have shown better antibacterial activity against *S. aureus* than compound **4**.

According to the literature (Khammar and Djeddi, 2012), the antimicrobial activity of the genus *Centaurea* is mainly attributed to a high content of sesquiterpene lactones. Three sesquiterpene lactones (13-acetylsolstitialin A, centaurepensin and chlorojanerin) isolated from the aerial parts of *Centaurea solstitialis* L. ssp. *solstitialis* showed inhibitory activity against standard *S. aureus* with identical MIC values of 16 µg/mL, respectively (Özçelik et al., 2009).

Concerning antibacterial activity against *A. baumannii* (Supplementary Information), the isolated compounds **1** to **6** exhibited no activity (all MIC and MBC values are above 62.5 µg/mL). These differences observed between antibacterial activities against *S. aureus* and *A. baumannii* can be mainly ascribed to differences in the cell wall structure of Gram-positive and Gram-negative bacteria (Maddison et al., 2008). While the cell wall of Gram-positive bacteria (*S. aureus*), which consist predominantly of peptidoglycan, allows for hydrophobic molecules to easily pass through the cells, the complex outer membrane of Gram-negative bacteria (*A. baumannii*) is almost totally impermeable to hydrophobic molecules (Nazzaro et al., 2013).

**Supplementary Table 2.** Reduction in the number of *A. baumannii,* MIC and MBC after treatment with *C. ragusina* isolated compounds as compared to the corresponding control.

| *Acinetobacter baumannii* DURN | | | | | | |
| --- | --- | --- | --- | --- | --- | --- |
|  | % reduction (μg mL^-1^ ) | | | | MIC (μg mL^-1^ ) | MBC (μg mL^-1^ ) |
| compounds | 2500 | 1250 | 125 | 62.5 |  |  |
| 1 | x | x | x | 8.4 | >62.5 | >62.5 |
| 2 | x | x | x | 16.0 | >62.5 | >62.5 |
| 3 | x | x | x | 14.1 | >62.5 | >62.5 |
| 4 | x | x | x | 12.2 | >62.5 | >62.5 |
| 5 | x | x | x | 15.0 | >62.5 | >62.5 |
| 6 | x | x | x | 12.2 | >62.5 | >62.5 |
| Values represent mean of 3 replicates. SD < 5%; nd – not detected, x – not measured. Ampicillin MIC <0.12 µg mL^-1^. Negative control is given in Experimental. | | | | | | |

2. UV/Vis experiments and CD experiments

The ctDNA was dissolved in Na cacodylate buffer, *I*=0.05 mol dm^-3^, pH 7.0 and additionally sonicated and filtered through a 0.45 mm filter (Chaires et al., 1982). DNA concentration was determined spectroscopically as the concentration of phosphates (Chargaff and Lipshitz, 1953).

The measurements were performed by adding aliquots of DMSO stock solutions to the buffer solution (pH = 7.0, *I* = 0.05 mol dm^-3^, sodium cacodylate buffer, DMSO content of the final solutions <0.6%). Under the experimental conditions used (concentration of compounds ≤ 3 × 10^-5^ mol dm^-3^ for UV/Vis and CD) the absorbance intensities of compounds were proportional to their concentrations. For UV/Vis experiments spectrophotometric titrations were performed in aqueous buffer solution by adding portions of polynucleotide solution into the solution of the studied compound. For CD experiments the titrations were done by adding portions of compound stock solution into the solution of the polynucleotide. CD titrations were done in 1cm path quartz cuvettes, with instrument parameters: standard sensitivity, scanning speed of 200 nm/min. Buffer background was subtracted from each spectrum, while each spectrum was the result of 3-5 accumulations.

**Supplementary Figure 1.** UV/Vis spectra of flavonoids (chrysin (compound **1**), oroxylin A (compound **2**) and hispidulin (compound **3**)), c = 1 × 10^-5^ M, Na-cacodylate buffer, pH=7.0, *I*=0.05 M)

The UV/Vis changes of all isolated flavonoids with ctDNA were too small (Δ Abs ≤ 0.04) for the accurate calculation of binding constants (Supplementary, Figure S2). In line with these findings, flavonoids also showed only a small decrease of CD spectra of ctDNA at 275 nm indicating weak interactions with ctDNA (Figure S3). Among isolated sesquiterpenes and flavonoids, only **5** (Figure S4) exhibited significant changes in CD titrations, a small decrease of positive CD band of ctDNA at 275 nm accompanied by minor bathochromic shift (2 nm) and a moderate increase of the negative CD signal at 245 nm.

a) b)

**Supplementary Figure 2.** a) Changes in UV/Vis spectrum of oroxylin A (compound **2**) (*c*= 2 × 10^-5^ mol dm^-3^) upon titration with ctDNA (*c*= 1.0 × 10^-5^ - 1.3× 10^-3^ mol dm^-3^); b) Dependence of oroxylin A absorbance at λ_max_ = 348 nm on c(ctDNA), at pH 7.0, sodium cacodylate buffer, *I* = 0.05 mol dm^-3^; the UV/Vis changes of compound **2** with ctDNA were too small (Δ Abs < 0.02) for the accurate calculation of binding constant.

**Supplementary Figure 3.** CD titration of ctDNA (*c* = 3.0 × 10^-5^ mol dm^-3^) with oroxylin A (compound **2**) at molar ratios ***r*** = [compound] / [polynucleotide] (pH 7.0, buffer sodium cacodylate, *I* = 0.05 mol dm^-3^).

**Supplementary Figure 4.** CD titration of ctDNA (*c* = 3.0 × 10^-5^ mol dm^-3^) with ragusinin (compound **5)** at molar ratios *r* = [compound] / [polynucleotide] (pH 7.0, buffer sodium cacodylate, *I* = 0.05 mol dm^-3^).

3. Isothermal titration calorimetry (ITC) experiments

The reference cell was filled with an aqueous buffer solution containing 0.3% DMSO. During the titration of compound **5** with ctDNA, one aliquot of 2 μL, 11 aliquots of 8 μL, 5 aliquots of 16 μL, 1 aliquot of 20 μL and 2 aliquots of 32 μL of the ctDNA (c = 1.5 × 10^-3^ mol dm^-3^) were injected from a rotating syringe (307 rpm) equilibrated at 25.0 °C into the isothermal cell containing 1.4406 mL of compound **5** (c = 3 × 10^-5^ mol dm^-3^). The spacing between each injection was 300 s. The initial delay before the first injection was 1600 s.

Blank experiments were carried out to determine the heats of dilution of compound **5**. All solutions used for ITC experiments were degassed under vacuum (0.64 bar, 10 min) prior to use to eliminate air bubbles. Each injection generated a heat burst curve (P in µW versus time). The data were imported to Origin 7.0 and the area under each peak was determined by integration to evaluate the heat associated with the injection. The data were corrected for heats of dilution. The resulting data were analyzed by using the Origin 7.0 software according to the model based on a single set of identical binding sites to estimate the binding constants (*K*_a_), the binding stoichiometry (N) and the enthalpy of binding (∆_r_*H*^o^). The reaction Gibbs energies (∆_r_*G*^0^) were calculated by using the following equation: ∆_r_*G*^o^ = -RTln(*K*_a_). Entropic contribution to the binding Gibbs energy was calculated by the equation: T∆_r_*S*^o^ = ∆_r_*H*^o^ - ∆_r_*G*^o^.

4. Cytotoxic activity

*Crystal violet (CV) assay*

Experiments were carried out in 96-wells microtiter plates. 1 × 10^4^ cells/250 μL of the medium was applied to each well (Ivanković et al., 2015). Twenty-four hours after the seeding, the cells reached subconfluence. The growth medium was replaced with the fresh one and 25 μL of sample (extract, fraction or compound) solution was added to the culture. The concentration of CRE and *C. ragusina* L. fractions was 0.06 mg/mL whilst isolated pure compounds were tested at two concentrations, i.e. 10 µM and 5 µM. Stock solutions of the samples were prepared with dimethyl sulfoxide (DMSO) and stored at -20 °C. All cell lines were incubated with the samples for 24 hours with 0.1% DMSO. Control cells were incubated in RPMI medium without addition of the studied samples but with 0.1% of DMSO. The 0.1% DMSO concentration in the medium did not have any significant effect on cell proliferation.

Stock solutions of the samples were prepared with dimethyl sulfoxide (DMSO) and stored at -20 °C. All cell lines were incubated with the samples for 24 hours with 0.1% DMSO. Control cells were incubated in RPMI medium without addition of the studied samples but with 0.1% of DMSO. The 0.1% DMSO concentration in the medium did not have any significant effect on cell proliferation.

After 24 h, crystal violet staining was performed to measure cell survival (Ivanković et al., 2015). In short, cells were fixed by the addition of a 3% solution of formalin for 15 min, washed with deionized water and dried in air. Then, cells were stained with 0.1% crystal violet for 20 min followed by extensive washing with deionized water and drying overnight. The dye was removed from the cells using a 10% solution of acetic acid and then absorbance was measured at 540 nm using a microplate reader. The absorbance at 540 nm is proportional to the number of surviving cells. The mean absorbance value of control cells was considered as 100% survival and the treated sample survival percentages were calculated by comparing the absorbance of treated samples (A_treated_) with a mean absorbance of the control (A_control_) (Cell survival in % = (A_treated_/A_control_) x 100). Each experiment was done in quadruplicate.


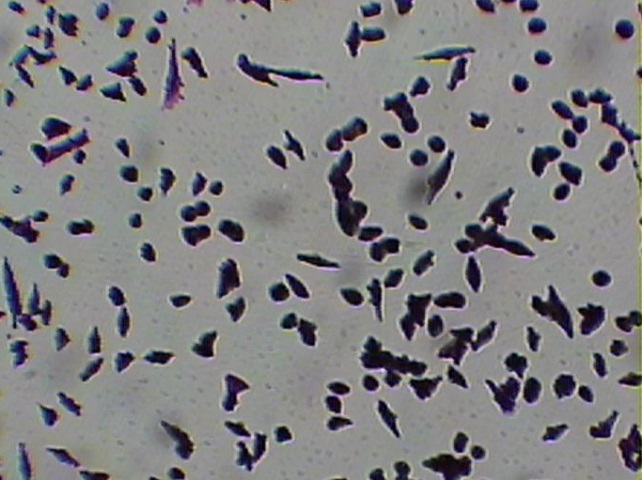

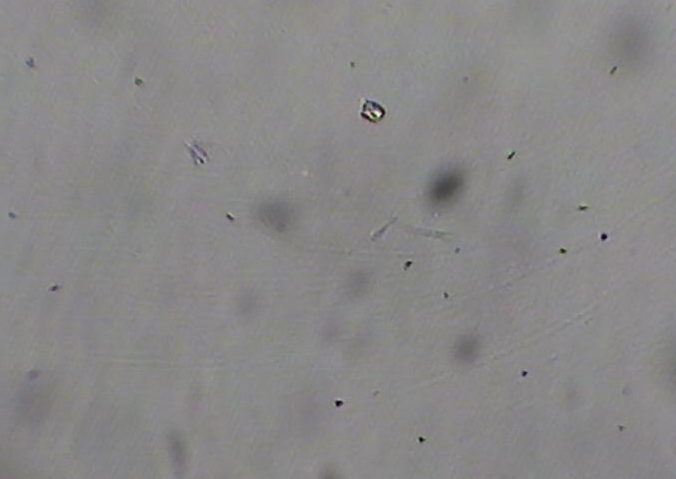


a) b)

**Supplementary Figure 5**. Photomicrographs of a) fixed control SCCVII cells and b) SCCVII cells treated with compound **5**, final concentration, c_(5)_ = 10 µM.

*(3-(4,5-dimethylthiazol-2-yl)-2,5-diphenyltetrazolium bromide) tetrazolium reduction (MTT) assay*

In short, the cells were seeded into 96-well tissue culture plates. The next day different concentrations of studied compound were added to each well in quadruplicate. Upon 72 h incubation at 37 °C, the medium was aspirated, and the 3-(4,5-dimethylthiazol-2-yl)-2,5-diphenyl-tetrazolium bromide dye (Sigma-Aldrich) was added. Three hours later, the formed formazan crystals were dissolved in DMSO, the plates were mechanically agitated for 5 min and the optical density at 570 nm was determined on a microtiter plate reader (Awareness Technology Inc., USA). Experiments were performed at least three times. The percentage of cell survival for each tested concentrations of the compound was calculated according to the absorption value of non-treated, control cells that was set as 100%.

5. Experiments with antioxidants

Tempol and salubrinal were purchased by Santa Cruz Biotechnology (Dallas, USA), dissolved in DMSO and kept by -20 °C. Trolox was purchased by Sigma-Aldrich, dissolved in water and kept by -20 °C. MTT assay was used for measurement of cell survival. The method is described under Material and Methods.





**Supplementary Figure 6.** The cells were either pre-treated for two hours with 12.5 μM salubrinal (A), 0.1 mM tempol (B) or 40 μM trolox (C). After that, the cells were treated with different concentrations of ragusinin (compound **5**). 72 h after MTT assay was performed. All experiments were performed at least three times.

References:

Chaires, J.B., Dattagupta, N. and Crothers, D.M. (1982). Studies on interaction of anthracycline antibiotics and deoxyribonucleic acid: equilibrium binding studies on interaction of daunomycin with deoxyribonucleic acid. Biochemistry. 21:3933-3940.

Chargaff, E., and Lipshitz, R. (1953). Composition of Mammalian Desoxyribonucleic Acids. J Amer. Chem. Soc. 75:3658-3661.

Facey, P.C., Peart, P.C., and Porter, R.B.R. (2010). The antibacterial activities of mikanolide and its derivatives. West Indian Med. J. 59:249-252.

Ivanescu, B., Miron, A., and Corciova, A. (2015). Sesquiterpene lactones from *Artemisia* genus: biological activities and methods of analysis. J. Anal. Methods Chem, 2015:247685. doi: 10.1155/2015/247685.

Khammar, A., and Djeddi, S. (2012). Pharmacological and Biological Properties of some *Centaurea* Species. Eur. J. Sci. Res. 84:398-416.

Maddison, J. E., Page, S. W., and Church, D. B. (BVSc.), Small Animal Clinical Pharmacology, 2nd Ed. Edinburgh; London: Elsevier Saunders, 2008; pp 157-168.

Nazzaro, F., Fratianni, F., De Martino, L., Coppola, R., and De Feo, V. (2013). Effect of Essential Oils on Pathogenic Bacteria. Pharmaceuticals, 6:1451-1474.

Özçelik, B., Gürbüz, I., Karaoglu, T., and Yeşilada, E. (2009). Antiviral and antimicrobial activities of three sesquiterpene lactones from *Centaurea solstitialis* L. ssp. *Solstitialis*, Microbiol. Res. 164:545-552.
